# MetF‐Dependent Methionine Biosynthesis Is Required for *Mycobacterium tuberculosis* Survival and In Vivo Persistence

**DOI:** 10.1002/mbo3.70370

**Published:** 2026-08-04

**Authors:** Ikue Tosa, Tomoki Kitahara, Katsuki Takebe, Masaaki Nakayama, Yuko Ito, Akihito Nishiyama, Shintaro Seto, Hotaka Kawai, Hiharu Inoue, Yuki Fukada, Yuki Nishiya, Hitoshi Nagatsuka, Takayuki Wada, Manabu Ato, Sohkichi Matsumoto, Naoya Ohara

**Affiliations:** ^1^ Department of Oral Microbiology Okayama University Graduate School of Medicine, Dentistry and Pharmaceutical Sciences Okayama Japan; ^2^ Advanced Research Center for Oral and Craniofacial Sciences, Dental School Okayama University Okayama Japan; ^3^ Department of Bacteriology, School of Medicine Niigata University Niigata Japan; ^4^ Department of Dental Pharmacology Okayama University Graduate School of Medicine, Dentistry and Pharmaceutical Sciences Okayama Japan; ^5^ Present Address: Microbiology, Division of Oral Pathobiological Science, Faculty and Graduate School of Dental Medicine Hokkaido University Hokkaido Japan; ^6^ Department of Pathophysiology and Host Defense The Research Institute of Tuberculosis, Japan Anti‐Tuberculosis Association Tokyo Japan; ^7^ Department of Basic Mycobacteriosis Nagasaki University Graduate School of Biomedical Sciences Nagasaki Japan; ^8^ Department of Oral Pathology and Medicine Okayama University Graduate School of Medicine, Dentistry and Pharmaceutical Sciences Okayama Japan; ^9^ Graduate School of Human Life and Ecology Osaka Metropolitan University Osaka Japan; ^10^ Department of Orthodontics Okayama University Graduate School of Medicine, Dentistry and Pharmaceutical Sciences Okayama Japan; ^11^ Department of Oral and Maxillofacial Reconstructive Surgery Okayama University Graduate School of Medicine, Dentistry and Pharmaceutical Sciences Okayama Japan; ^12^ Osaka International Research Center for Infectious Diseases Osaka Metropolitan University Osaka Japan; ^13^ Leprosy Research Center, National Institute of Infectious Diseases, Japan Institute for Health Security Tokyo Japan; ^14^ Department of Bacteriology Osaka Metropolitan University Graduate School of Medicine Osaka Japan; ^15^ Division of Research Aids Hokkaido University Institute for Vaccine Research and Development Hokkaido Japan; ^16^ Department of Medical Microbiology, Faculty of Medicine Universitas Airlangga Surabaya Indonesia; ^17^ Research Center for Intestinal Health Science Okayama University Okayama Japan

**Keywords:** bacterial growth, in vivo essentiality, MetF, methionine biosynthesis pathway, mouse infection model, mycobacterium tuberculosis

## Abstract

Methionine biosynthesis is essential for *Mycobacterium tuberculosis*, but the in vivo relevance of individual enzymes remains unclear. We performed transposon sequencing in *Mycobacterium bovis* BCG under methionine‐free conditions and identified *metF* as a major fitness determinant. To define enzyme‐specific functions, ΔmetE and ΔmetF mutants were generated in *M. tuberculosis* and evaluated using *in vitro* growth assays and a murine infection model. The ΔmetE mutant showed vitamin B_12_‐dependent growth in minimal medium but maintained normal growth in mice, indicating that its *in vitro* requirement does not translate to an in vivo defect. In contrast, ΔmetF exhibited strict methionine auxotrophy *in vitro* and completely failed to survive or persist in murine lungs and spleens. Structural modeling showed that MetF is distinct from human homologs, supporting selective inhibition. These findings demonstrate that MetF, but not MetE, is indispensable for *M. tuberculosis* survival in vivo and identify MetF as a promising metabolic drug target.

## Graphical abstract

MetF is essential for both *in vitro* and in vivo growth of *Mycobacterium tuberculosis*, whereas MetE, previously considered essential *in vitro*, is dispensable in vivo. Given the importance of methionine and folate metabolism as drug targets, MetF represents a promising candidate, highlighting the need for in vivo validation.

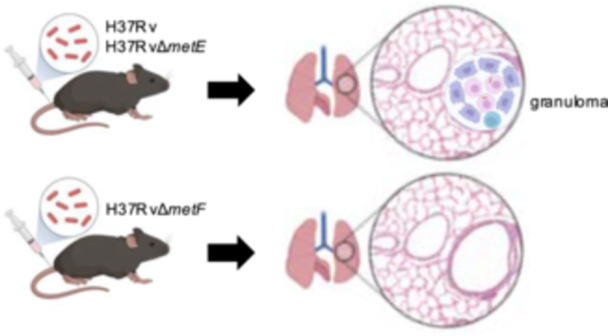

## 1 Introduction

Tuberculosis (TB) is a lethal infectious disease caused by *Mycobacterium tuberculosis*, transmitted through airborne droplets. According to the World Health Organization (WHO), TB remains a global health concern, with over 10 million new cases and more than 1 million deaths reported annually(Global Tuberculosis Report 2025 2025). Current TB treatment primarily relies on chemotherapy, with rifampicin and isoniazid serving as cornerstone drugs. However, the emergence of multidrug‐resistant TB (MDR‐TB)—resistant to both rifampicin and isoniazid—and extensively drug‐resistant TB (XDR‐TB)—resistant to rifampicin (and may also be resistant to isoniazid), and that is also resistant to at least one fluoroquinolone (levofloxacin or moxifloxacin) and to at least one other Group A drug (bedaquiline or linezolid)—poses serious clinical challenges(Global Tuberculosis Report 2025 2025). Furthermore, the prolonged duration and complexity of current treatment regimens underscore the urgent need for the development of new anti‐tuberculosis drugs that are effective against drug‐resistant strains, exhibit high efficacy, and can shorten the treatment period.

One promising strategy in anti‐TB drug development is targeting the metabolic pathways of *M. tuberculosis*. A notable example is para‐aminosalicylic acid (PAS), a classic anti‐TB drug that targets the folate biosynthesis pathway and has regained attention for its efficiency against drug‐resistant TB(Desai and Joshi 2018). The methionine biosynthesis pathway, which is directly linked to folate metabolism, has been identified as essential for *M. tuberculosis* survival(Berney et al. 2015) and is therefore considered a promising target for novel drug development.

**Figure A1:**
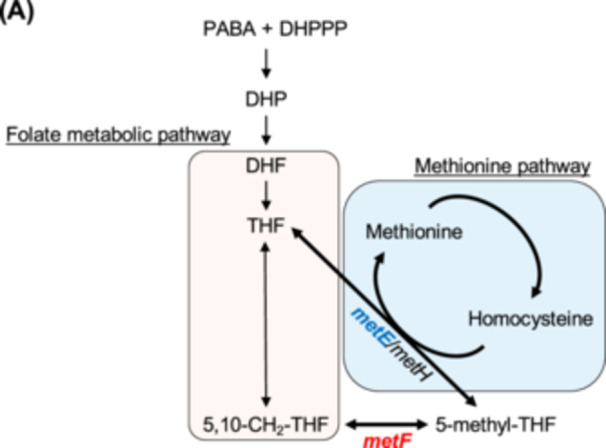
Schematic representation of the folate biosynthetic pathway in *Mycobacterium tuberculosis*. A diagram illustrating the proposed folate biosynthetic pathway in *M. tuberculosis*, highlighting the enzymatic steps involved in the conversion of precursors into folate derivatives. PABA: para‐aminobenzoic acid, DHPPP: 6‐hydroxymethyl‐7,8–dihydropterin‐pyrophosphate, DHP: 7,8–dihydropteroate, DHF: 7,8–dihydrofolate, THF: Tetrahydrofolate.

Methionine is an essential amino acid required for protein synthesis and for the production of *S*‐adenosylmethionine, a universal methyl donor involved in numerous cellular processes. *M. tuberculosis* synthesizes methionine *de novo* via a pathway that involves two distinct methionine synthases: MetE, which is vitamin B_12_‐independent, and MetH, which is vitamin B_12_‐dependent. The enzyme MetF (5,10‐methylenetetrahydrofolate reductase) catalyzes the formation of 5‐methyltetrahydrofolate (5‐methyl THF), the methyl donor necessary for the conversion of homocysteine to methionine (Figure A1).

To systematically identify genetic determinants of methionine metabolism beyond these known synthases, we performed transposon sequencing (Tn‐seq) using *Mycobacterium bovis* BCG (BCG) cultured in methionine‐supplemented and methionine‐free media. Comparative analysis of transposon insertion frequencies revealed hundreds of genes exhibiting differential fitness between the two conditions. Gene set enrichment analysis indicated enrichment in sulfur metabolism and amino acid biosynthesis pathways, and notably, the *metF* gene showed a pronounced growth defect under methionine‐free conditions, suggesting its critical role in methionine biosynthesis.

Previous studies have demonstrated that methionine biosynthesis in *M. tuberculosis* is tightly regulated by vitamin B_12_ availability. MetE functions as a vitamin B_12_‐independent methionine synthase, whereas MetH requires vitamin B_12_ as a cofactor. Recent studies have shown that *M. tuberculosis* lacks endogenous vitamin B_12_ biosynthesis but can acquire vitamin B_12_ from the host environment, thereby enabling metabolic switching between MetE‐ and MetH‐dependent methionine synthesis. Consistent with this model, MetE‐deficient strains can be rescued by exogenous vitamin B_12_ under defined culture conditions (Campos‐Pardos et al. 2024; Warner et al. 2007). Recent studies have also highlighted MetE as an immunogenic antigen and a potential vaccine candidate, further emphasizing the biological importance of methionine metabolism in *M. tuberculosis* (Almujri et al. 2025; De Voss et al. 2025; Iacobino et al. 2024).

Although recent studies have clarified the roles of vitamin B_12_, MetE, and MetH in methionine metabolism and demonstrated that host‐derived vitamin B_12_ can compensate for loss of MetE during infection, the contribution of MetF to bacterial survival and persistence in vivo remains largely unexplored. Therefore, we constructed *M. tuberculosis* mutants lacking either *metE* or *metF* and systematically compared their phenotypes under defined nutritional conditions *in vitro* and during murine infection. Our results demonstrate that while *metE* is dispensable in vivo due to metabolic compensation, *metF* is essential and structurally distinct from its human counterpart, representing a promising target for the development of next‐generation anti‐TB therapeutics.

## 2 Materials and Methods

### 2.1 Bacterial Strains and Culture Conditions

*Escherichia coli* strain DH5α (Takara Bio, Shiga, Japan) was cultured at 37°C aerobically in lysogeny broth (LB) medium (Nacalai Tesque, Kyoto, Japan).

BCG Tokyo‐172 strain and *M. tuberculosis* H37Rv strain were grown at 37°C aerobically in Middlebrook 7H9 broth (Becton, Dickinson and Company, Franklin Lakes, NJ, USA) supplemented with 10% albumin‐dextrose‐catalase (ADC), 0.2% glycerol, and 0.05% Tween 80 (Sigma‐Aldrich, St. Louis, MO, USA) or on Middlebrook 7H10 agar (Becton, Dickinson and Company) plates with 10% oleic acid‐albumin‐dextrose‐catalase (OADC) and 0.5% glycerol. Sauton agar plate was also used, composed of 0.5 g/L KH_2_PO_4_, 0.5 g/L MgSO_4_, 2.0 g/L citric acid monohydrate, 4.0 g/L l‐asparagine, 60 mL/L glycerol, 0.05 g/L ammonium iron (III) citrate (pH 7.4), and 1.5% agar. The medium was supplemented with methionine (50 µg/mL) for Tn‐seq.

Carbenicillin (50 μg/mL; FUJIFILM Wako Pure Chemical, Osaka, Japan), kanamycin (20 μg/mL; Meiji Seika Pharma, Tokyo, Japan) or hygromycin B (50 μg/mL; FUJIFILM Wako Pure Chemical) was added to the medium when required.

### 2.2 Genomic DNA Extraction From Mycobacterial Strains

Genomic DNA was extracted from BCG Tokyo‐172 and *M. tuberculosis* strains using a modified chloroform/methanol and phenol‐based protocol(Belisle and Sonnenberg 1998). Briefly, bacterial cells were frozen and treated with chloroform/methanol (2:1, v/v) to remove lipids from the cell wall. They were then treated with rLysozyme^TM^ Solution (EMD Millipore Corp., Burlington, MA, USA), followed by SDS and proteinase K. Protein contaminants were removed by extraction with phenol:chloroform:isoamyl alcohol (25:24:1).

### 2.3 Tn‐Seq

The positions of Tn insertion in the genomic DNA of the constructed Tn libraries were determined using a method previously described (Long et al. 2015) with modification. In brief, genomic DNA was shared into approximately 500 bp fragments by ultrasonication using the Covaris S220 system. The resulting DNA fragments were subjected to end repair, A‐tailing and ligation to an adaptor sequence. Fragments containing transposon‐genomic junctions were selectively enriched during the initial PCR with primers corresponding to the transposon and the adaptor sequences, followed by second heminested PCR primers. The resulting amplicons were then subjected to 75 bp paired‐end sequencing on an Illumina NextSeq. 500 and raw sequence data were exported to fastq files for further analysis.

### 2.4 Bioinformatics

Sequence data were analyzed using TRANSIT(DeJesus et al. 2015; Ioerger 2022). Reads were mapped to the BCG Tokyo 172 strain AP010918.1 genome using the TPP pipeline implemented in TRANSIT. Insertion counts obtained from TPP were analyzed using the resampling algorithm in TRANSIT to compare gene fitness between the growth in the methionine‐supplemented and methionine‐free media. For resampling analysis, insertions in the central 90% of each gene were considered and a LOSS correlation for genome positional bias was performed. Adjusted *p* values for multiple comparisons were estimated using the Benjamini–Hochberg procedure to maintain false‐discovery rate at lower 5%.

Genes identified as showing significantly GD under either condition were subjected to GSEA(Subramanian et al. 2005) based on KEGG pathway annotations implemented in clusterProfiler (version 4.16.0; Yu et al. 2012). A significance cutoff of adjusted *p* < 0.25 was used for enrichment detection.

### 2.5 Strain Construction

To construct the *metE* and *metF* knockout alleles, the upstream and downstream regions of each gene were amplified from the *M. tuberculosis* H37Rv genome. The coding sequence of the hygromycin resistance gene was amplified from pBabe‐Hygro (Cell Biolabs, San Diego, CA, USA). These fragments were assembled into the pUC19 vector using Gibson Assembly Master Mix (New England Biolabs, Ipswich, MA, USA). All PCR amplifications were performed with PrimeSTAR GXL DNA Polymerase (Takara Bio). Primer sequences are listed in Table A1.

Bacterial cells were suspended in electroporation buffer consisting of 272 mM mannitol, 2 mM sodium phosphate buffer (pH 7.1), and 0.05% Tween 80. Cells were transformed with the plasmid pJV53 (Addgene, plasmid # 26904), which encodes the Che9c mycobacteriophage recombinase(van Kessel and Hatfull 2007).

**Figure A2:**
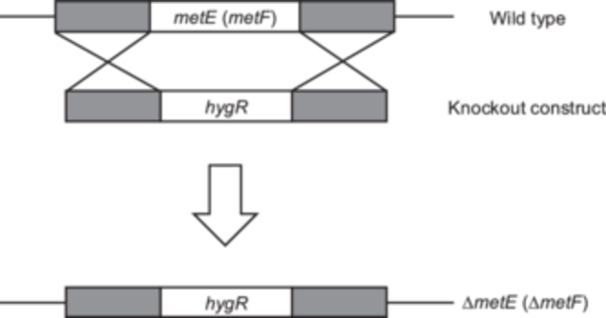
Genetic constructs used for the generation of ΔmetE and ΔmetF mutant strains. Schematic representation of the gene disruption strategy for *metE* and *metF* loci in *M. tuberculosis*. hygR: hygromycin resistance gene.

The pJV53 transformed *M. tuberculosis* were then cultured in 7H9 medium containing kanamycin and 0.2% acetamide overnight to induce expression of the Che9c recombinase. After induction, cells were suspended in electroporation buffer and electroporated with the *metE* or *metF* knockout construct. Each construct contained the 5′ and 3′ flanking regions of *metE* or *metF*, respectively, positioned on either side of the hygromycin resistance cassette (Figure A2). Following electroporation, hygromycin‐resistant colonies were isolated and screened as candidate ∆metE and ∆metF mutants.

### 2.6 Whole Genome Sequencing and Data Analysis

Whole genome sequencing was performed by Rhelixa Inc. (Tokyo, Japan) using a PCR‐free library preparation method. Genomic DNA libraries were prepared with the NEBNext Ultra II DNA Library Prep Kit (New England Biolabs), and sequencing was conducted on the Illumina NovaSeq X Plus platform. Paired‐end reads of 150 bp x 2 (PE150) were generated, yielding approximately 1 Gb of data per sample, corresponding to an average of 6.7 million reads (3.3 million read pairs) per sample.

Short reads were mapped to the reference genome sequence of the parental strain *M. tuberculosis* H37Rv (GenBank: AL123456.3) using Burrows‐Wheeler Alignment tool(Li and Durbin 2009) (version 0.7.19‐r1273). Integrative Genomics Viewer(Robinson et al. 2011) (version 2.19.7) was used for the visualization.

### 2.7 Bacterial Growth

Bacterial suspensions of *M. tuberculosis* H37Rv, ΔmetE, and ΔmetF strains were streaked onto Sauton or Middlebrook 7H10 agar plates and incubated at 37°C for up to 119 days. Where indicated, methionine (50 µg/mL), vitamin B_12_ (10 µg/mL), or homocysteine (50 µg/mL) were supplemented.

For growth kinetics in liquid culture, bacterial suspensions were adjusted to an OD_590_ of 5.0 × 10^−2^ and incubated at 37°C. ΔmetE strains were cultured in the presence of 0, 1, 10, or 100 ng/mL of vitamin B_12_, while ΔmetF strains were cultured with 0, 1, 5, or 25 μg/mL of methionine. Bacterial growth was monitored by measuring OD_590_ up to 42 days.

### 2.8 Mice

Female C57BL/6 J mice (8 weeks old) were purchased from CLEA Japan Inc. (Tokyo, Japan). All animal procedures, including housing and experimental protocols, were reviewed and approved by the Institutional Animal Care and Use Ethics Committee of Niigata University (SA01342). Mice were cared for in accordance with the institutional guidelines for the use of laboratory animals. All experiments were conducted in compliance with the ARRIVE guidelines.

### 2.9 Infection Experiment

Mice were intravenously infected via the tail vein with 1 ×10^8^ colony‐forming units (CFU) of *M. tuberculosis* H37Rv (wild‐type), ΔmetE, or ΔmetF strains. At 3, 28, and 42 days post‐infection, animals were euthanized, and lungs and spleens were harvested for downstream analyses.

### 2.10 CFU Enumeration

Lung and spleen tissues were homogenized in sterile saline, and the resulting homogenates were serially diluted and plated on Middlebrook 7H10 agars. Plates were incubated at 37°C for 4 weeks, after which CFUs were enumerated, and bacterial loads were calculated per organ.

### 2.11 Histology

Excised lungs were fixed in 10% neutral‐buffered formalin, embedded in paraffin, and sectioned at 5 μm thickness. Sections were stained with hematoxylin and eosin (H&E) and Ziehl–Neelsen stain using standard protocols. Images were acquired using a BX53 microscope (Olympus, Tokyo, Japan) or a BZ‐X710 microscope (Keyence, Osaka, Japan).

### 2.12 Immunohistochemical Staining (IHC)

IHC was performed using F4/80 antibody (CST; D2S9R, Cell Signaling Technology, Danvers, MA, USA). Sections were deparaffinized in xylene for 15 min, rehydrated in a graded ethanol solution. The sections were blocked with 1.2% hydrogen peroxide/methanol solution to inhibit endogenous peroxidase activity. Antigen retrieval was done according to the manufacturer's instructions. After antigen retrieval, the sections are blocked with 10% normal serum for 15 min at room temperature and incubated with primary antibodies at 4°C overnight. Signals were enhanced by the avidin‐biotin complex method (Vector Laboratories, Newark, CA, USA). Color development was performed with DAB (Histofine DAB substrate), and sections were counterstained with Myer's hematoxylin. Staining results were observed under an optical microscope (BX53, Olympus).

### 2.13 Structural Analysis

Amino acid sequences were retrieved from the UniProt database (Human MTHFR: P42898; *M. tuberculosis* MTHFR [MetF]: O53506.) Sequence alignments were performed using ClustalW software (https://www.genome.jp/tools-bin/clustalw)(Thompson et al. 1994). The human MTHFR structure was obtained from the Protein Data Bank (PDBID: 6FCX). The predicted structure of *M. tuberculosis* MTHFR was generated using AlphaFold2(Jumper et al. 2021). Protein structures were visualized and analyzed with PyMOL 2.5 (open‐source version).

### 2.14 Statistical Analysis

Statistical analyses were conducted using Prism 9 (GraphPad Software, La Jolla, CA, USA). Comparisons between two groups were performed using two‐tailed unpaired Student's *t*‐tests, and comparisons among multiple groups were assessed by one‐way ANOVA followed by Tukey's post hoc test. *p* < 0.05 was considered statistically significant. *p < 0.05, **p < 0.01, and ***p < 0.001. Results are expressed as mean ± SEM, and all data points represent biological replicates.

## 3 Results

### 3.1 Gene Fitness of BCG During Growth in Methionine‐Containing Medium Revealed By Transposon Sequencing

**Figure 1:**
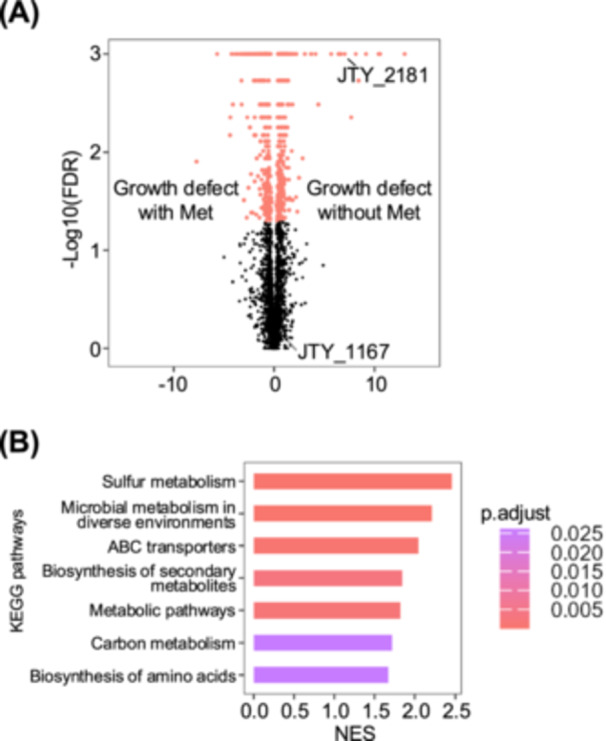
Gene fitness of *M. bovis* grown in media without and with methionine. (A) Volcano plot of differential gene fitness between growth in media without and with methionine. Red plots represent genes with FDR < 0.05. In total, 302 and 286 genes were identified as growth defect (GD) in media without and with methionine, respectively. (B) Gene set enrichment analysis (GSEA) of gene required for growth. Significantly enriched KEGG pathways are shown. Positive normalized enrichment scores (NES) indicate pathways enriched in the medium with methionine relative to that without methionine.

To evaluate the gene fitness of BCG during growth in methionine‐supplemented medium, we compared the frequencies of transposon (Tn) insertions between bacteria grown in media with or without methionine (50 µg/mL). Four independent Tn mutant libraries of the BCG Tokyo 172 were generated via transduction with the mycobacteriophage phAE180 carrying the Himar1 transposon(Kriakov et al. 2003). Genomic DNA was extracted from each Tn mutant library and subjected to transposon sequencing (Tn‐seq). Gene fitness in the methionine‐supplemented medium relative to the methionine‐free condition was determined using the resampling algorithm implemented in TRANSIT(DeJesus et al. 2015). We identified 302 and 286 genes exhibiting growth defects (GD) in the methionine‐free and methionine‐supplemented media, respectively (Figure 1A and Supplementary Table A2). Gene set enrichment analysis (GSEA) based on KEGG pathways was performed using these GD genes (Figure 1B). GD genes identified in the methionine‐free medium were enriched in pathways related to sulfur metabolism, microbial metabolism in diverse environments, ABC transporters, and amino acid biosynthesis. Although no significantly enriched pathways were detected among GD genes from the methionine‐supplemented condition, *adoK* (adenosine kinase), *sigB* (sigma factor B), and *thyA* (thymidylate synthase) were included among these genes. Notably, the *metF* gene (JTY_2181) exhibited a strong growth defect in the methionine‐free condition.

### 3.2 Construction of *metE* and *metF* Deletion Mutants

**Figure A3:**
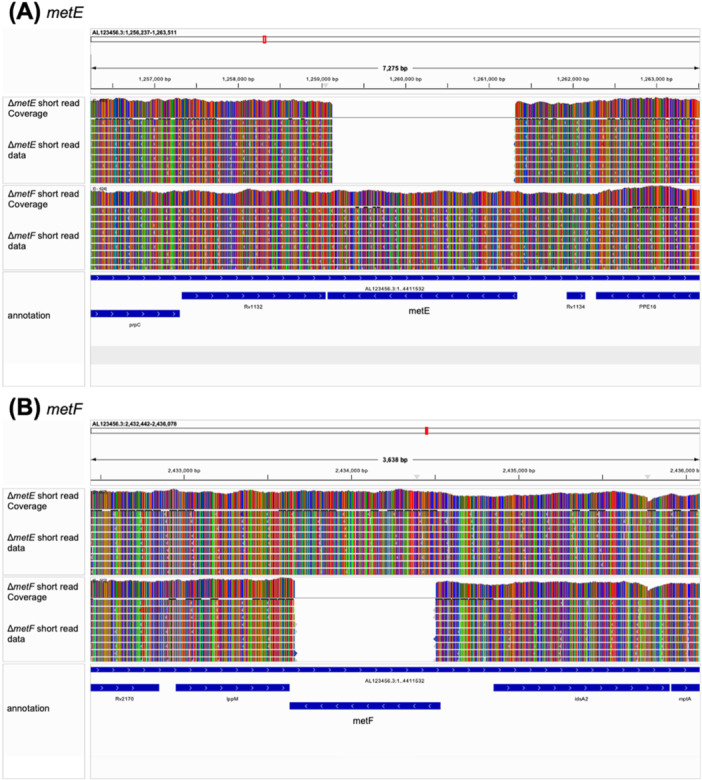
Whole genome sequencing of ΔmetE and ΔmetF strains**.** The region around the (A) *metE* and (B) *metF* genes of *M. tuberculosis* H37Rv is shown when the short reads of the ΔMetE and ΔMetF strains are mapped to the reference genome sequence (GenBank: AL123456.3). (Top) The range of the genome sequence of H37Rv. (Middle) Mapping coverage and short read mapping of the short reads of ΔMetE and ΔMetF strains. The maximum coverage of this region is 577 and 562, in the mapping of ΔMetE and ΔMetF, respectively. (Bottom) Gene annotation of *M. tuberculosis* H37Rv.

Deletion mutants of *metE* and *metF* in *M. tuberculosis* H37Rv were generated via double‐crossover allelic exchange using a pJV53‐derived system, in which the target genes were replaced with a hygromycin resistance cassette (Figure A2). To confirm the successful deletion of *metE* and *metF* in the ΔmetE and ΔmetF strains, whole‐genome sequencing was performed. Short reads were mapped to the reference genome of the parental H37Rv strain. As expected, a marked reduction in read coverage was observed over the *metE* and *metF* loci, and the hygromycin resistance gene was found to be inserted exclusively at the corresponding deleted regions (Figure A3).

### 3.3 Growth Phenotypes of ΔmetE and ΔmetF Mutants Under Various Nutritional Conditions

**Figure 2:**
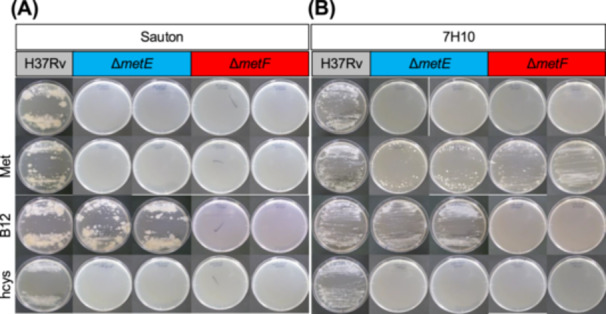
Colony formation of ΔmetE and ΔmetF strains. Colony formation of *M. tuberculosis* H37Rv (wild‐type), ΔmetE, and ΔmetF strains on (A) minimal Sauton agar and (B) nutrient‐rich 7H10 agar. Plates were incubated at 37°C and monitored for up to 119 days. Met: methionine, B12: vitamin B_12_, hcys: homocysteine.

To examine the growth characteristics of the ΔmetE and ΔmetF mutant strains, we first assessed colony formation on agar media. Two types of media were used to compare different nutritional environments: minimal Sauton medium and nutrient‐rich 7H10 medium. To evaluate the impact of methionine biosynthesis pathway intermediates on bacterial growth, four supplementation conditions were tested for each medium: no supplement, methionine, vitamin B_12_, or homocysteine. Cultures were incubated for up to 119 days. Consistent with previous reports (Campos‐Pardos et al. 2024), the ΔmetE mutant failed to grow in the absence of vitamin B_12_ but was rescued by vitamin B_12_ supplementation (Figure 2A,B). In addition, colonies of the ΔmetE strain were observed with methionine supplementation after prolonged incubation (119 days) on nutrient‐rich 7H10 agar (Figure 2B), although no colonies were visible at the 23‐day time point (data not shown). In contrast, ΔmetF strain failed to grow under any condition tested on minimal Sauton agar, (Figure 2A) and showed growth only when methionine was added, and not with vitamin B_12_ or homocysteine on nutrient‐rich 7H10 agar (Figure 2B). Homocysteine supplementation did not rescue growth in either mutant under any condition. These findings are consistent with a model in which vitamin B_12_‐dependent MetH activity compensates for the loss of MetE, whereas MetF is required for methionine biosynthesis irrespective of vitamin B_12_ availability.

### 3.4 Dose‐Dependent Effects of Vitamin B_12_ and Methionine on the Growth of ΔmetE and ΔmetF Mutants

**Figure 3:**
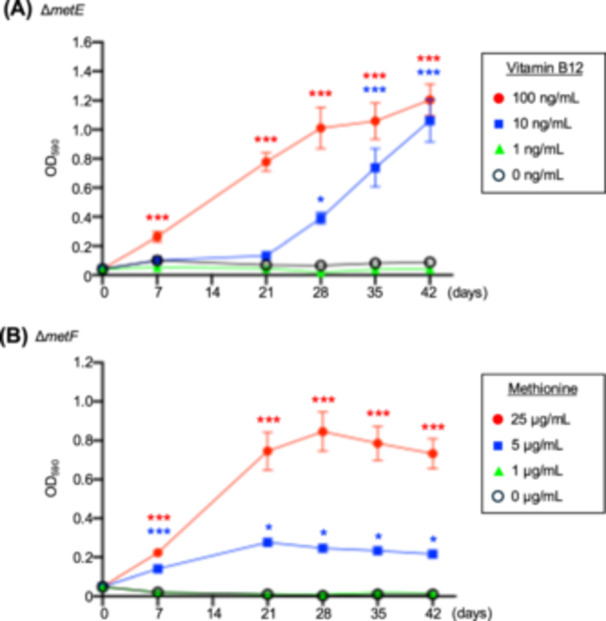
Growth kinetics of ΔmetE and ΔmetF mutant strains under nutrient supplementation. Growth curves of (A) ΔmetE cultured in 7H9 medium supplemented with 0, 1, 10, or 100 ng/mL of vitamin B_12_ and (B) ΔmetF cultured with 0, 1, 5, or 25 µg/mL of methionine. All cultures were initiated at an OD_590_ of 0.05 and incubated at 37°C for up to 42 days (*n* = 3–5). Asterisks indicate statistically significant differences compared to the unsupplemented control at each time point. Statistical analysis was performed using one‐way ANOVA followed by Tukey's post hoc test.

To determine the minimum concentrations required for growth rescue, we cultured the ΔmetE and ΔmetF strains in 7H9 liquid medium supplemented with increasing concentrations of vitamin B_12_ (0, 1, 10, 100 ng/mL) or methionine (0, 1, 5, 25 μg/mL), respectively. OD_590_ was measured at days 7, 21, 28, 35, and 42 to assess the impact of supplement concentration on bacterial proliferation. For the ΔmetE strain, supplementation with 100 ng/mL vitamin B_12_ led to a significant increase in OD_590_ as early as day 7 compared to the unsupplemented control (0 ng/mL). By day 28, the 10 ng/mL group also showed significantly higher OD_590_ values than the control, and at all time points (days 7, 21, and 28), OD_590_ values in the 100 ng/mL group were significantly higher than those in the 10 ng/mL group (*p* < 0.001), indicating a clear dose‐dependent enhancement of growth. In contrast, 1 ng/mL vitamin B_12_ failed to support growth, suggesting that this concentration is insufficient for metabolic rescue (Figure 3A). Similarly, the ΔmetF strain exhibited significantly increased OD_590_ values at all time points (days 7–42) when cultured with 5 or 25 μg/mL methionine compared to the control. Furthermore, at each time point, 25 μg/mL methionine consistently supported significantly greater growth than 5 μg/mL (*p* < 0.001), confirming a concentration‐dependent effect. No growth was observed with 1 μg/mL methionine, indicating that this level is below the threshold required for growth (Figure 3B). These results suggest that the growth defects of ΔmetE and ΔmetF can be rescued in a dose‐dependent manner by vitamin B_12_ and methionine, respectively, and that sufficient concentrations of these metabolites are essential for restoring proliferation in the absence of endogenous biosynthetic enzymes.

### 3.5 Attenuated In Vivo Growth of the ΔmetF Mutant in a Mouse Infection Model

**Figure 4:**
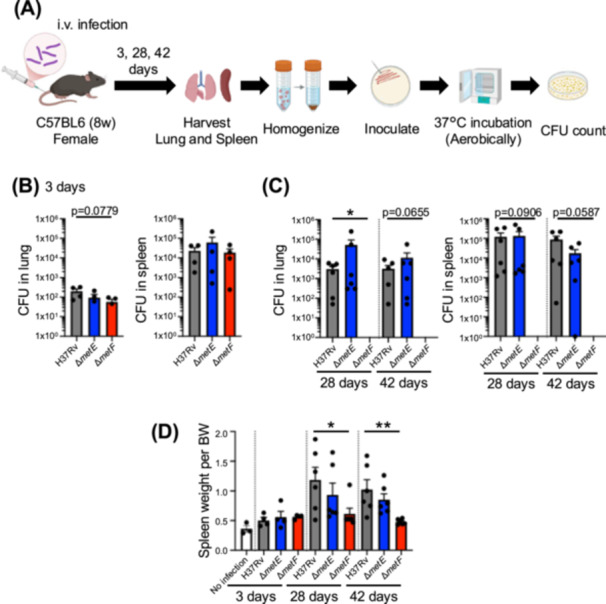
Bacterial burden in lungs and spleens of mice infected with ΔmetE and ΔmetF strains. (A) Schematic representation of the intravenous infection protocol. C57BL/6 J mice were infected with *M. tuberculosis* H37Rv (wild‐type), ΔmetE, or ΔmetF strains via the tail vein. Lung and spleen tissues were harvested at 3, 28, and 42 days post‐infection for downstream analyses. (B) Quantification of bacterial burden in lungs and spleens at 3 days post‐infection to confirm successful establishment of infection. Tissues were homogenized, serially diluted, and plated on Middlebrook 7H10 agar for CFU enumeration (*n* = 4). (C) Quantification of bacterial burden in lungs and spleens at 28 and 42 days post‐infection (*n* = 6). (D) Quantification of spleen weight normalized to body weight (*n* = 3–6). BW: body weight. Statistical analysis was performed using Student's *t*‐test.

**Figure 5:**
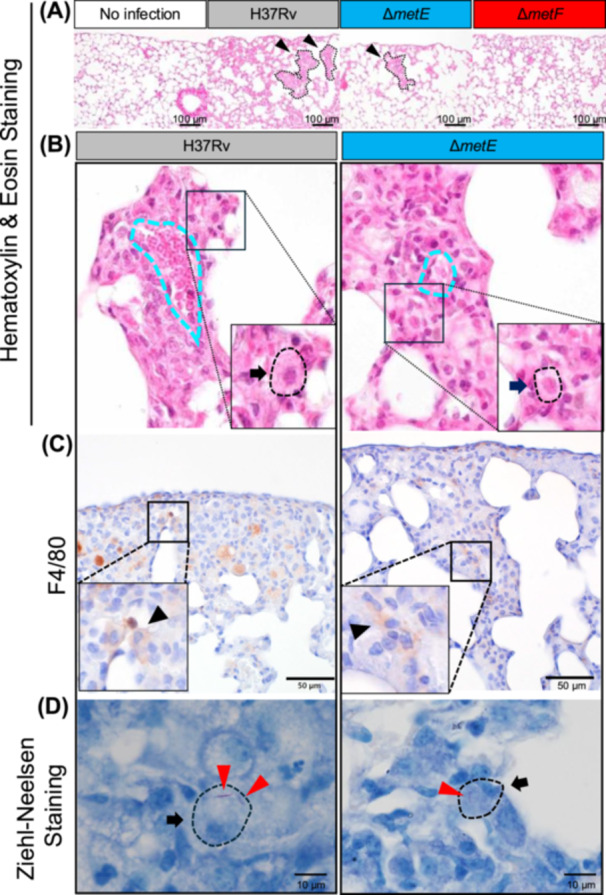
Histopathological and bacteriological evaluation of lung tissues following infection with ΔmetE and ΔmetF strains. (A) Representative hematoxylin and eosin (H&E)–stained sections show granuloma formation in lungs infected with H37Rv and ΔmetE strains (black arrowheads), whereas no granuloma formation was observed in lungs infected with the ΔmetF strain. (B) Higher magnification views of granulomas in H37Rv‐ and ΔmetE‐infected lungs reveal central capillaries lined by endothelial cells (outlined in light blue) and the presence of foamy macrophages (black arrows), both displaying comparable histological features. (C) Immunohistochemical staining for F4/80 confirmed that these cells were macrophages (black arrows). (D) Ziehl–Neelsen staining demonstrated the presence of acid‐fast bacilli (red arrowheads) within foamy macrophages (black arrows) inside granulomas of both H37Rv‐ and ΔmetE‐infected mice.

To evaluate the in vivo growth capacity of the ΔmetE and ΔmetF mutants, 8‐week‐old C57BL/6 J mice were intravenously infected with *M. tuberculosis* H37Rv, ΔmetE, or ΔmetF strains. At 3 days post‐infection, lungs and spleens were harvested, homogenized, plated on agar, and incubated under aerobic conditions at 37°C to determine colony‐forming units (CFUs). This confirmed the establishment of infection for all strains, as CFUs were detected from both organs in each group. Notably, the ΔmetF strain exhibited a trend toward reduced CFUs in the lung compared to H37Rv, although the difference did not reach statistical significance (Figure 4A,B). To assess bacterial persistence and growth in vivo, CFUs were also measured at 28 and 42 days post‐infection. At both time points, the ΔmetE strain maintained bacterial loads in the lungs and spleen comparable to those of H37Rv strain. In contrast, the ΔmetF strain was undetectable in both organs at days 28 and 42, indicating a failure to persist or replicate in vivo (Figure 4C). In mice infected with the ΔmetF strain, spleen weight normalized to body weight was significantly lower at both 28 and 42 days post‐infection compared to the H37Rv‐infected group. These findings suggest that infection‐induced splenomegaly seen in H37Rv‐infected mice was largely suppressed in the ΔmetF‐infected group (Figure 4D). Histological analysis of lung tissue at day 42 revealed granuloma formation in mice infected with H37Rv and ΔmetE strains (Figure 5A, black arrowheads). Within the granulomas, capillaries lined by endothelial cells (Figure 5B, outlined in light blue) were present in the central region, and foamy cells (Figure 5B, black arrows) were observed. The foamy cells were positive for F4/80 (Figure 5C, black arrows), it indicated that those cells were macrophages; both groups displayed histologically identical structures. Ziehl–Neelsen–positive acid‐fast bacilli (Figure 5D, red arrowheads) were detected within the foamy macrophages inside the granulomas (Figure 5D, black arrows). In contrast, no granuloma formation was observed in mice infected with the ΔmetF strain (Figure 5A). These findings suggest that while MetE is dispensable for in vivo persistence under certain conditions, MetF is essential for survival and replication of *M. tuberculosis* in the host.

## 4 Discussion

Given the essentiality of methionine for *M. tuberculosis*, its biosynthetic pathway has been studied as a potential antimicrobial target. In particular, the conversion of homocysteine to methionine has been considered a critical step. Recent studies have established that *M. tuberculosis* relies on host‐derived vitamin B_12_ and dynamically regulates the MetE/MetH branch of methionine synthesis according to vitamin availability (Campos‐Pardos et al. 2024; Warner et al. 2007). In this study, we further examined the growth capacity of the MetE‐deficient strain under defined nutritional conditions. On Sauton agar plate, which is relatively nutrient‐poor, growth occurred only with vitamin B_12_ supplementation. In contrast, growth on 7H10 agar plate—containing albumin and other components—was supported by either vitamin B_12_ or methionine. The dose‐dependent response to vitamin B_12_ suggests that MetH, a vitamin B_12_‐dependent enzyme, can compensate for the absence of MetE. Importantly, MetE‐deficient strains also grew in vivo. Although their growth in mice cannot be explained by serum vitamin B_12_ levels alone (approximately 500 pg/mL (Ghosh et al. 2016)), they likely utilize both MetH‐dependent synthesis and circulating methionine. Consistently, experiments using 7H10 agar plate demonstrated that the MetE‐deficient strain could grow in the presence of methionine. This indicates that, in vivo, methionine uptake likely works together with MetH‐dependent methionine synthesis to support the proliferation of *M. tuberculosis*. Because serum vitamin B_12_ concentrations in humans (118–1158 pg/mL (Galukande et al. 2011)) are comparable to those in mice, MetE may function as an *in vitro* target but is unlikely to serve as an effective therapeutic target in vivo.

Importantly, our findings extend previous studies by demonstrating a clear distinction between conditional and absolute essentiality within the methionine biosynthesis pathway. While disruption of MetE can be compensated during infection, likely through host‐derived vitamin B_12_ and MetH‐mediated methionine synthesis, loss of metF could not be bypassed under either *in vitro* or in vivo conditions. MetF generates N5‐methyl THF, the methyl donor required for the conversion of homocysteine to methionine. Although MetF‐deficient strains were rescued by methionine in a concentration‐dependent manner, neither homocysteine nor vitamin B_12_ supported their proliferation. This underscores the indispensable role of N5‐methyl THF in methionine synthesis. Notably, MetF‐deficient strain failed to grow in the lungs or spleens of infected mice. Although serum methionine levels in mice (10 ng/mL(Jeon et al. 2018)) exceed the minimum concentration required *in vitro*, only a fraction is likely bioavailable. When bacterial proliferation is minimal, enhanced immune recognition and phagocytic clearance may further limit persistence. Indeed, at 5 ng/mL methionine—the concentration approximating the levels accessible to *M. tuberculosis in vivo*—the bacteria did not enter the logarithmic growth phase. Therefore, unlike the MetE‐deficient strain, the MetF‐deficient strain is unable to grow in vivo and consequently considered a promising drug target for tuberculosis treatment.

**Figure A4:**
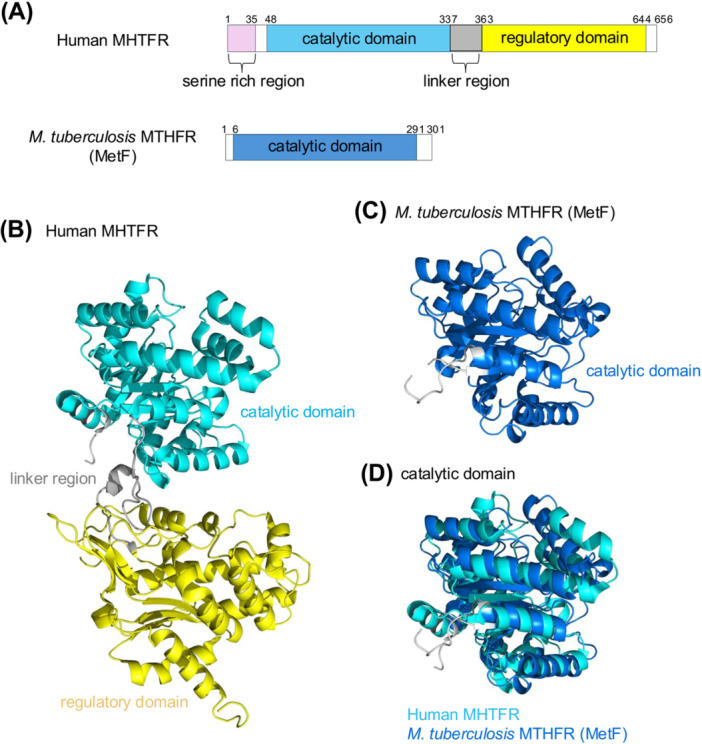
Structural analysis of MetF and MTHFR. (A) Schematic representation of human and *M. tuberculosis* methylenetetrahydrofolate reductase (MTHFR). The catalytic domain of human MTHFR is shown in cyan, the linker region in gray, and the regulatory domain in yellow. The catalytic domain of *M. tuberculosis* MTHFR (MetF) is shown in marine. (B) Crystal structure of the catalytic domain, linker region and regulatory domain of human MTHFR (PDBID: 6FCX). Domain colors correspond to those in panel (A). (C) Predicted full‐length structure of *M. tuberculosis* MTHFR. Domain color corresponds to those in panel (A). (D) Superimposition of the catalytic domain of *M. tuberculosis* MetF and human MTHFR. Domain colors correspond to those in panel (A).

Structural analysis further supports the potential of MetF as a drug target. MTHFR enzymes can be classified into four distinct types based on domain composition(Froese et al. 2018). While human MTHFR contains a serine‐rich region, catalytic domain, and regulatory domain, *M. tuberculosis* MTHFR (MetF) consists solely of the catalytic domain. Superimposition of the predicted *M. tuberculosis* MTHFR (MetF) structure with the catalytic domain of human MTHFR (PDB ID: 6FCX) revealed only 14.95% sequence identity and a root‐mean‐square deviation (RMSD) of the backbone atoms was 3.852 Å, demonstrating low structural similarity (Figure A4). This suggests that selective inhibition of bacterial MetF may be achieved without significant off‐target effects on the host enzyme. Additionally, prior studies have reported that mutations in MetF (R159N and L214A) reduce enzymatic activity and increase susceptibility of *M. tuberculosis* to antifolates such as PAS and SMX(Yu et al. 2022). Taken together, our findings indicate that inhibition of MetF could not only directly impair bacterial survival but also potentiate the efficacy of existing drugs in combination therapies.

A limitation of study is the absence of *in vitro* macrophage infection assays. Although our mouse experiments demonstrated severe attenuation of the MetF‐deficient strain, the cellular mechanisms underlying this defect remain unknown. In particular, whether MetF loss directly compromises intracellular survival has yet to be clarified. Future studies employing macrophage infection models will be essential to define the cell‐intrinsic requirements for methionine metabolism during intracellular infection and determine whether MetF inhibition sensitizes *M. tuberculosis* to immune pressures.

## 5 Conclusion

MetF is indispensable for in vivo survival, structurally distinct from the host enzyme, and therefore represents a compelling target for the development of new anti‐tuberculosis therapeutics.

## Author Contributions

**Ikue Tosa:** investigation, formal analysis, writing – original draft. **Tomoki Kitahara:** investigation, formal analysis. **Katsuki Takebe:** investigation, formal analysis, writing – original draft. **Masaaki Nakayama:** investigation, formal analysis. **Yuko Ito:** investigation, formal analysis. **Akihito Nishiyama:** investigation, formal analysis. **Shintaro Seto:** methodology, formal analysis, writing – review and editing. **Hotaka Kawai:** methodology, investigation, formal analysis, writing – review and editing. **Hiharu Inoue:** formal analysis, writing – review and editing. **Yuki Fukada:** Formal analysis. **Yuki Nishiya:** formal analysis, writing – review and editing. **Hitoshi Nagatsuka:** resources, supervision. **Takayuki Wada:** supervision, methodology. **Manabu Ato:** writing – review and editing, resources, funding acquisition. **Sohkichi Matsumoto:** investigation, methodology, resources, writing – review and editing, funding acquisition, supervision. **Naoya Ohara:** conceptualization, methodology, resources, writing – review and editing, supervision, funding acquisition.

## Ethics Statement

All animal procedures, including housing and experimental protocols, were reviewed and approved by the Institutional Animal Care and Use Ethics Committee of Niigata University (SA01342). Mice were cared for in accordance with the institutional guidelines for the use of laboratory animals. All experiments were conducted in compliance with the ARRIVE guidelines.

## Conflicts of Interest

The authors declare no conflicts of interest.

## Supporting information


Supporting File

## Data Availability

The data that support the findings of this study are openly available in DDBJ at https://www.ddbj.nig.ac.jp/index.html, reference number DRR760804‐DRR760811 and DRR751162‐DRR751166. Requests for further information and resources should be directed to and will be fulfilled by the lead contact, Naoya Ohara (oharan@md.okayama-u.ac.jp). The sequencing data have been deposited in the DDBJ Sequence Read Archive (DRA) and can be accessed through the accession number DRR760804‐DRR760811 and DRR751162‐DRR751166.

## 1

**Table A1:** Primers used in this study.

Name	Sequence (5′ to 3′)	Application	Product size
metE_UF	CGGAATTAGCTTGGTACGGGATCCGATCATGGTCTGCTTTCACCTGGGG	*metE* upstream region	864 bp
metE_UR	GAGGTGACCGCGTCGCTGCACAACATGGTC		
metE_DF	AAAGGGTTGACGACGTACAGGCTGGGTCAC	*metE* downstream region	879 bp
metE_DR	GCCTATGGAAAAACGCCATGCATGGCTCACCACGGTTTTACAGCGTC		
hygR_EF	GTTGTGCAGCGACGCGGTCACCTCCCGATATTCCTTTGCCCTCGGACG	*hygR* cassette for *metE* knockout	1075 bp
hygR_ER	GCCTGTACGTCGTCAACCCTTTATGAAAAAGCCTGAACTCACCGCG		
metF_UF	CGGAATTAGCTTGGTACGGGATCCCACCGACATCATCCGCGGCTACCAC	*metF* upstream region	908 bp
metF_UR	TCACCTGCCGAGCCGGGCAAGCCGGACTAG		
metF_DF	CAGCTCCAGCGCGATCGTGTTGAGGGTCAC	*metF* downstream region	882 bp
metF_DR	GCCTATGGAAAAACGCCATGCATGTCGAGGTATTGCCCGCCCAGCACC		
hygR_FF	GGCTTGCCCGGCTCGGCAGGTGACCGATATTCCTTTGCCCTCGGACG	*hygR* cassette for *metF* knockout	1076 bp
hygR_FR	CTCAACACGATCGCGCTGGAGCTGATGAAAAAGCCTGAACTCACCGCG		
